# Emotional and Behavioral Impairment and Comorbid Eating Disorder Symptoms in Adolescents with Obesity: A Cross-Sectional Study

**DOI:** 10.3390/jcm13072068

**Published:** 2024-04-03

**Authors:** Anna Guerrini Usubini, Michela Bottacchi, Adele Bondesan, Francesca Frigerio, Nicoletta Marazzi, Gianluca Castelnuovo, Alessandro Sartorio

**Affiliations:** 1 Psychology Research Laboratory, Istituto Auxologico Italiano, Istituto di Ricovero e Cura a Carattere Scientifico (IRCCS), 20145 Milan, Italy; m.bottacchi@auxologico.it (M.B.); gianluca.castelnuovo@auxologico.it (G.C.); 2 Experimental Laboratory for Auxo-Endocrinological Research, Istituto Auxologico Italiano, Istituto di Ricovero e Cura a Carattere Scientifico (IRCCS), 28824 Piancavallo-Verbania, Italy; a.bondesan@auxologico.it (A.B.); f.frigerio@auxologico.it (F.F.); sartorio@auxologico.it (A.S.); 3 Experimental Laboratory for Auxo-Endocrinological Research, Istituto Auxologico Italiano, Istituto di Ricovero e Cura a Carattere Scientifico (IRCCS), 20145 Milan, Italy; n.marazzi@auxologico.it; 4 Department of Psychology, Catholic University of Milan, 20123 Milan, Italy

**Keywords:** obesity, adolescents, eating disorders, strengths and difficulties questionnaire, depression, anxiety

## Abstract

**Background**: The current study aims to assess the psychological conditions of Italian adolescents with obesity seeking an in-hospital multidisciplinary body weight reduction program, by exploring their psychological adjustment, emotional states, and co-occurring eating disorder symptoms. **Methods**: The study involved ninety-two consecutive Italian adolescents with obesity (31 males, 61 females), with a mean age ± SD: 16.4 ± 1.1 years and body mass index (BMI): 38.3 ± 6.04 kg/m^2^). The Strengths and Difficulties Questionnaire (SDQ), Beck Depression Inventory (BDI), State-Trait Anxiety Inventory (STAI), and Eating Attitude Test-26 (EAT-26) were used for the evaluations. Differences between genders, degrees of obesity (Group 1 = BMI SDS 2–2.99 and Group 2: BMI SDS > 3), and those with or without eating disorder symptoms (Group 1: EAT-26 ≤ 20 and Group 2: EAT-26 > 20) were explored. **Results**: The results showed that females reported higher scores on the Emotional Symptoms, Prosocial Behaviors, Total Difficulties, and Total Impact subscales of the SDQ, the BDI, both subscales of the STAI, and the Bulimia subscales of the EAT-26 than males, independently from the degrees of obesity. Participants with eating disorder symptoms (Group 2: EAT-26 > 20) showed higher scores on the Emotional Symptoms and Total Difficulties subscales of the SDQ, the BDI, and both subscales of the STAI than those of Group 1 (EAT-26 ≤ 20). **Conclusions**: The study explores the psychological conditions of adolescents with obesity. The results can inform appropriate treatment approaches for the management of obesity in developmental age groups, which not only take into account the medical and physical aspects of obesity, but also the behavioral, emotional, and social difficulties expressed by adolescents, in addition to specific eating disorder symptoms.

## 1. Introduction

In the last decades, obesity in adolescents has risen dramatically worldwide. In European countries, one in three school-aged children, one in four adolescents, and almost 60% of the adult population are overweight or obese [1].

Obesity is a complex disease and is attributed to the interplay of biological, psychological, and environmental factors. The presence of obesity in adolescence increases the risk of prediabetes, type 2 diabetes, hypertension, non-alcoholic fatty liver disease (NAFLD), metabolic syndrome, as well as the development of sleep disorders, such as obstructive sleep apnea (OSA), refs. [2,3], and menstrual irregularities in adolescent girls.

Obesity not only impacts the physical health of adolescents but also their psychological well-being. Adolescents with obesity may experience many psychological issues including depression, anxiety, social isolation, low self-esteem, behavioral problems, body dissatisfaction, and reduced quality of life [4]. Adolescents with obesity can be bullied and stigmatized at school, with pervasive implications for their emotional and physical health [5].

In addition, obesity can often co-occur with eating disorders, in particular binge eating disorder (BED) and bulimia nervosa (BN) [6,7]. BED is characterized by recurrent binge eating episodes occurring at least once a week for the past 3 months, and it is associated with marked distress. The same binge eating episodes, followed by unhealthy compensatory behaviors to prevent weight gain (such as self-induced vomiting, use of laxatives, and excessive physical activity), are the main symptoms of BN [8]. Similar to other EDs, BN is mainly characterized by self-evaluations that are excessively influenced by body weight and shape [9].

Weight and shape concerns, disordered eating attitudes, and behaviors including unhealthy weight control behaviors or binge eating are elevated in adolescents with obesity and can contribute to several areas of psychological impairment. For instance, in some studies, adolescents with obesity who experienced uncontrolled eating were at higher risk of depression and body dissatisfaction than adolescents with obesity without uncontrolled eating [10].

The co-occurrence between obesity and EDs requires attention, since individuals with obesity and comorbid EDs are at higher risk for several complications than those with one or the other condition alone [11,12].

Despite the fact that remarkable efforts in examining the association between obesity and many psychological comorbidities continue to be made, significant gaps in knowledge still remain. In particular, less is known about the pediatric population, which remains less studied than adults.

The present study is aimed at assessing the psychological adjustment, emotional states (depression and anxiety in particular), and the prevalence of co-occurring eating disorder symptoms in a sample of Italian adolescents with obesity seeking a multidisciplinary body weight reduction program as a treatment for obesity. In this exploration, the role of gender, and different degrees of obesity have been considered. Finally, possible differences between patients with obesity with and without ED symptoms in psychological adjustment and emotional states have been analyzed.

## 2. Materials and Methods

### 2.1. Participants and Procedures

The study involved ninety-two consecutive Italian adolescents with obesity (31 males, 61 females), with a mean age ± SD: 16.4 ± 1.1 years and body mass index (BMI): 38.3 ± 6.04 kg/m^2^). Participants were recruited at the Division of Auxology, Istituto Auxologico Italiano IRCCS, Piancavallo (VB), a third-level clinical center for the rehabilitation of individuals with obesity and eating disorders. Inclusion criteria were as follows: (1) Italian citizenship; (2) between 11 and 17 years of age; and (3) a BMI > 97th centile, according to the Italian growth charts [13]. Participants were excluded if they had any form of physical or mental impairment that could compromise their participation in the study.

After being informed about the research and after obtaining both written informed consent to participate from their parents and assent from the young patients, participants were screened for participation in the study. A clinical interview was conducted by an independent psychologist with expertise in clinical psychology to assess inclusion/exclusion criteria. All patients who were asked to participate met all the inclusion criteria. Once enrolled, participants were asked to answer self-report questionnaires at the beginning of a 3-week body weight reduction program. Participants were recruited at the time of admission to the hospital and the questionnaires were administered within the first 48 h of hospitalization.

The current study was approved by the Ethical Committee of Istituto Auxologico Italiano, IRCCS, Milan, Italy (approval number: 2021_01_26_03). The research was carried out according to the Declaration of Helsinki and its advancements.

### 2.2. Measures

Weight and height were measured by the internal medical staff. Standing height was determined by a Harpenden Stadiometer (Holtain Limited, Crymych, Dyfed, UK). Weight was measured to the nearest 0.1 kg using an electronic scale (RoWU 150, Wunder Sa.bi., Trezzo sull’Adda, Italy). Body mass index (BMI) was calculated according to the proper formula: kg/m^2^.

Clinical data were obtained via the administration of the Italian-validated versions of the following self-report questionnaires.

The Italian version [14] of the Strengths and Difficulties Questionnaire (SDQ) [15] was used as a screening tool for assessing emotional and behavioral problems. It is composed of 25 items, rated on a three-point Likert scale (0 = not true, 1 = somewhat true, or 2 = certainly true), and addresses five subscales: Emotional Symptoms, Conduct Problems, Hyperactivity and Inattention, Peer Problems, and Prosocial Behavior. All but the last of these subscales are summed to generate a Total Difficulties score, with higher scores reflecting greater difficulties. On the contrary, higher scores on the Prosocial Behavior subscale reflect more strength. Five additional items, addressing how difficulties upset or distress the child and interfere with home life, friendship, classroom learning, and leisure activities are summarized to produce the Total Impact score.

The Italian version [16] of the Beck Depression Inventory [17] was used to measure depressive symptoms. It is composed of 21 items and rated on a four-point Likert scale (from 0 to 3). For each item, participants are required to choose which sentence best describes how they have been feeling during the last two weeks. The total score ranges from 0 to 63, with higher scores reflecting higher levels of depression.

The State-Trait Anxiety Inventory [18], Italian version [19], was used to assess anxiety symptoms. It is composed of 40 items, rated on a four-point Likert scale, and divided into two scales: State Anxiety and Trait Anxiety. The total score ranges from 20 to 80, with higher scores indicating greater anxiety.

The Eating Attitude Test-26 (EAT-26) [20], Italian version [21], was used to assess eating disorder symptoms. It is composed of 26 items, rated on a 6-point Likert scale (0 = never, 5 = always), and addresses three subscales: Dieting, Bulimia, and Oral Control. The total score ranges from 0 to 78, and a score equal to or higher than 20 indicates that a subject may be at risk of an eating disorder. Higher scores reflect more severe symptomatology.

### 2.3. Statistical Analysis

Frequencies and percentages were calculated for categorical variables and means, and standard deviations were calculated for continuous variables. The normal distribution of the variables was assessed by skewness and kurtosis indices. To assess differences in males and females and different degrees of obesity in the SDQ, BDI, STAI, and EAT-26, a series of independent sample *t*-tests were conducted.

Initially, an analysis was performed on the total sample. Then, we explored the difference between males and females, and we also divided the sample into two subgroups of BMI SDS: Group 1 = BMI SDS 2–2.99 and Group 2: BMI SDS > 3. Finally, differences in the SDQ, BDI, and STAI into two subgroups according to the EAT-26 scores were explored: Group 1: EAT-26 ≤ 20 and Group 2: EAT-26 > 20. Independent sample *t*-tests were used. The critical alpha was set at 0.05, and Cohen’s d was used as a measure of the effect size.

## 3. Results

Originally, ninety-five adolescents with obesity fulfilled the inclusion criteria for the current study. Three subjects were subsequently excluded due to the incomplete compilation of one or more questionnaires. The final sample was composed of ninety-two consecutive Italian adolescents with obesity (31 males, 61 females), with a mean age ± SD: 16.4 ± 1.1 years and body mass index (BMI): 38.3 ± 6.04 kg/m^2^). Descriptive statistics of the study population are presented in Table 1.

Significant differences in the Emotional Symptoms (*p* < 0.001), Prosocial Behaviors (*p* = 0.030), Total Difficulties (*p* = 0.003), and Total Impact (*p* < 0.001) subscales of the SDQ were observed, with higher scores in females than males.

No significant differences between the two subgroups of BMI SDS in the SDQ were found.

Significant differences were found in the BDI (*p* < 0.001), and the State-Anxiety (*p* = 0.002) and Trait-Anxiety (*p* = 0.001) subscales of the STAI, with higher scores for females than males.

No significant differences between the two subgroups of BMI SDS in the BDI and STAI were found.

Significant differences in the Bulimia (*p* < 0.001) and Total (*p* = 0.021) subscales of the EAT-26 were found, with higher scores for females than males.

No significant differences between the two subgroups of BMI SDS in the EAT-26 were found.

The results are presented in Table 2.

Significant differences in the Emotional Symptoms (*p* < 0.001), Total Difficulties (*p* = 0.014) subscales of the SDQ, the BDI (*p* < 0.001), and the State-Anxiety (*p* < 0.001) and Trait-Anxiety (*p* < 0.001) subscales of the STAI were found, with higher scores in Group 2 than Group 1.

The results are presented in Table 3.

## 4. Discussion

Consistently with our line of research, the current study aimed at assessing the psychological conditions of Italian adolescents with obesity who were seeking an in-hospital multidisciplinary body weight reduction program, not only by highlighting their strengths and difficulties in their psychological adjustment [22], but also by exploring their emotional states (depression and anxiety in particular) and the prevalence of co-occurring eating disorder symptoms. In this exploration, the role of gender, different degrees of obesity, and the possible differences between those with and without eating disorder symptoms in psychological adjustment, depression, and anxiety symptoms were also analyzed. In this regard, our results show that females reported higher scores on the Emotional Symptoms, Prosocial Behaviors, Total Difficulties, and Total Impact subscales of the SDQ, the BDI, both subscales of the STAI, and the Bulimia subscale of the EAT-26 than males. All these results seem to be independent of the severity of obesity, which is suggested by the non-significant differences between the two subgroups of BMI-SDS.

These results expand upon those obtained by our research group in a previous study, which evaluated psychological adjustment in a sample of hospitalized adolescents with obesity using the SDQ. In that study, females reported significantly higher Emotional Symptoms, Peer Problems, Total Difficulties, and Total Impact scores on the SDQ than males [22], corroborating the hypothesis that female adolescents with obesity are more likely to show worse psychological conditions than males, including emotional problems, depression, and anxiety [23]. Fortunately, they also showed higher scores in Prosocial Behaviors than males, as previously reported in the literature [24], which represents a strength in their psychosocial functioning.

Depression and anxiety are common in adolescents with obesity [25,26], and are more common in females than males [27]. In the current study, significantly higher scores on the BDI and both subscales of the STAI were found in females than in males. Similar findings of greater anxiety and depression in female children than in male children with obesity (aged between 6 and 17 years) were reported by Lindberg and colleagues [28].

Eating disorder symptoms and obesity can co-occur. Taş Torun and colleagues [29] found that disordered eating behaviors were more common in adolescents seeking treatment for obesity than in the control group of normal-weight adolescents. In addition, Tronieri and colleagues explored gender differences in obesity and obesity-related mental health problems, highlighting a higher prevalence of eating disorders in women with obesity than in men [30]. Consistently, in our study, females reported higher scores of the Bulimia subscale of the EAT-26 than males. No other significant differences between genders in eating disorder symptoms were found.

The current study also inspected differences between participants with and without symptoms of eating disorders in psychological adjustment and depressive and anxiety symptoms.

By dividing the sample into two subgroups according to the EAT-26 score, we found that participants with eating disorder symptoms (Group 2: EAT-26 > 20) showed higher scores in the Emotional Symptoms and Total Difficulties subscales of the SDQ, the BDI, and both subscales of the STAI than Group 1 (EAT-26 ≤ 20). These results are in line with previous findings that indicated positive associations between EAT-26 scores and scores on the Emotional Symptoms and Total Difficulties subscales of the SDQ, thus suggesting that the higher the score on the EAT-26, the higher the score on the SDQ subscales. Similar findings were also obtained by Sparti and colleagues [31], who explored disordered eating among adolescents and found that those who obtained higher scores on the EAT-26 were more likely to show problems with functioning explored via the SDQ.

In general, our results seem to be in line with the evidence that adolescents with obesity are likely to experience exacerbated physical and psychological health issues [11].

This study is not free from limitations. This study is cross-sectional, with the lack of a control group and longitudinal measures, thus limiting possible causal conclusions. In addition, the sample was recruited in a single third-level clinical center, and this can limit the generalizability of the results for different subsets. Furthermore, the use of self-report measures to assess clinical and psychological parameters might carry the risk of biases, such as socially desirable responses. Future replications with a control group of normal-weight adolescents, the inclusion of additional measurements evaluating emotion dysregulation, and the inclusion of longitudinal data could improve the value of our results.

Despite the above limitations, the present study offers the opportunity to explore the psychological conditions of adolescents with obesity in a more in-depth manner and come to understand the underlying psychological determinants of obesity and their interactions in adolescents. The results of the study can inform appropriate treatment approaches for the management of obesity in developmental age groups, which not only take into account the medical and physical aspects of obesity, but also the behavioral, emotional, and social difficulties expressed by adolescents, in addition to specific eating disorder symptoms.

## Figures and Tables

**Table 1:** Descriptive statistics of the sample.

	*N*	M (SD)	Subgroups (*N*)	M (SD)
SDQ subscales				
Emotional Symptoms	92	4.97 (2.78)	M (31)	3.10 (2.30)
			F (61)	5.92 (2.51)
			Group 1 (42)	4.69 (2.83)
			Group 2 (50)	5.20 (2.73)
Conduct Problems	92	2.58 (1.92)	M (31)	2.00 (1.66)
			F (61)	2.00(2.04)
			Group 1 (42)	2.19 (1.67)
			Group 2 (50)	2.90 (2.06)
Hyperactivity–Inattention	92	4.42 (2.25)	M (31)	4.06 (2.14)
			F (61)	4.61 (2.30)
			Group 1 (42)	3.98 (1.98)
			Group 2 (50)	4.80 (2.41)
Peer Problems	92	3.76 (2.25)	M (31)	3.26 (2.38)
			F (61)	4.02 (2.16)
			Group 1 (42)	3.74 (2.23)
			Group 2 (50)	3.78 (2.29)
Prosocial Behaviors	92	8.18 (1.71)	M (31)	7.65 (1.70)
			F (61)	8.46 (1.66)
			Group 1 (42)	8.29 (1.49)
			Group 2 (50)	8.10 (1.89)
Total Difficulties	92	15.73 (6.98)	M (31)	12.77 (6.84)
			F (61)	17.23 (6.61)
			Group 1 (42)	14.60 (6.37)
			Group 2 (50)	16.68 (7.38)
Total Impact	92	2.54 (2.92)	M (31)	1.06 (1.84)
			F (61)	3.30 (3.08)
			Group 1 (42)	2.33 (2.68)
			Group 2 (50)	2.72 (3.12)
BDI Total	92	19 (13.3)	M (31)	12.3 (8.92)
			F (61)	22.4 (13.9)
			Group 1 (42)	19.1 (14.5)
			Group 2 (50)	18.9 (12.4)
STAI subscales				
State Anxiety	92	48 (13.3)	M (31)	42.2 (12.26)
			F (61)	51 (13)
			Group 1 (42)	46.5 (13.7)
			Group 2 (50)	49.3 (13)
Trait Anxiety	92	47.5 (13.1)	M (31)	41.5 (11.46)
			F (61)	50.5 (12.9)
			Group 1 (42)	46.6 (13.5)
			Group 2 (50)	48.1 (12.9)
EAT-26 subscales				
Dieting	92	8.98 (6.72)	M (31)	7.77 (4.27)
			F (61)	9.59 (7.64)
			Group 1 (42)	9.83 (7.95)
			Group 2 (50)	8.26 (5.47)
Bulimia	92	2.99 (4.12)	M (31)	1.16 (2.08)
			F (61)	3.92 (4.58)
			Group 1 (42)	2.93 (4.53)
			Group 2 (50)	3.04 (3.78)
Oral Control	92	1.45 (2.03)	M (31)	1.48 (1.84)
			F (61)	1.43 (2.13)
			Group 1 (42)	1.52 (1.95)
			Group 2 (50)	1.38 (2.11)
Total	92	13.04 (10.8)	M (31)	10.42 (6.20)
			F (61)	14.93 (12.31)
			Group 1 (42)	14.29 (13.04)
			Group 2 (50)	12.68 (8.61)

Note: M: males; F: females; Group 1: BMI SDS 2–2.99; Group 2: BMI SDS > 3; SDQ: Strengths and Difficulties Questionnaire; BDI: Beck Depression Inventory; STAI: State-Trait Anxiety Inventory; EAT-26: Eating Attitude Test-26.

**Table 2:** Differences between males and females and between Group 1 (BMI SDS 2-2.99) and Group 2 (BMI SDS > 3) on the SDQ, BDI, STAI and EAT-26.

	Subgroups (*N*)	Student’s *t*	*p*	Cohen’s d Effects Size
SDQ subscales				
Emotional Symptoms	M (31)	5.235	<0.001	1.155
	F (61)			
	Group 1 (42)	−0.876	0.383	−0.183
	Group 2 (50)			
Conduct Problems	M (31)	0.787	0.433	0.174
	F (61)			
	Group 1 (42)	−1.789	0.077	−0.374
	Group 2 (50)			
Hyperactivity–Inattention	M (31)	1.094	0.277	0.241
	F (61)			
	Group 1 (42)	−1.770	0.080	−0.370
	Group 2 (50)			
Peer Problems	M (31)	1.539	0.127	0.340
	F (61)			
	Group 1 (42)	−0.088	0.930	−0.018
	Group 2 (50)			
Prosocial Behaviors	M (31)	2.204	0.030	0.486
	F (61)			
	Group 1 (42)	0.517	0.606	0.108
	Group 2 (50)			
Total Difficulties	M (31)	3.020	0.003	0.666
	F (61)			
	Group 1 (42)	−1.435	0.155	−0.300
	Group 2 (50)			
Total Impact	M (31)	3.699	<0.001	0.816
	F (61)			
	Group 1 (42)	−0.631	0.530	−0.132
	Group 2 (50)			
BDI Total	M (31)	4.24	<0.001	0.870
	F (61)			
	Group 1 (42)	0.062	0.950	0.013
	Group 2 (50)			
STAI subscales				
State Anxiety	M (31)	3.13	0.002	0.691
	F (61)			
	Group 1 (42)	−1.009	0.315	−0.211
	Group 2 (50)			
Trait Anxiety	M (31)	3.30	0.001	0.728
	F (61)			
	Group 1 (42)	−0.543	0.588	−0.113
	Group 2 (50)			
EAT-26 subscales				
Dieting	M (31)	1.461	0.147	0.293
	F (61)			
	Group 1 (42)	1.120	0.266	0.234
	Group 2 (50)			
Bulimia	M (31)	3.965	<0.001	0.775
	F (61)			
	Group 1 (42)	−0.129	0.898	−0.026
	Group 2 (50)			
Oral Control	M (31)	−0.128	0.898	−0.028
	F (61)			
	Group 1 (42)	0.337	0.737	0.070
	Group 2 (50)			
Total	M (31)	2.340	0.021	0.4633
	F (61)			
	Group 1 (42)	0.707	0.482	0.147
	Group 2 (50)			

Note: M: males; F: females; Group 1: BMI SDS 2-2.99; Group 2: BMI SDS > 3; SDQ: Strengths and Difficulties Questionnaire; BDI: Beck Depression Inventory; STAI: State-Trait Anxiety Inventory; EAT-26: Eating Attitude Test-26.

**Table 3:** Differences between Group 1 (EAT-26 ≤ 20) vs. Group 2 (EAT-26 < 20) on the SDQ, BDI, and STAI.

		M (SD)	Student’s *t*	*p*	Cohen’s d Effect Size
SDQ subscales					
Emotional Symptoms	Group 1 (*n* = 74)	4.45 (2.67)	−3.934	<0.001	−1.034
	Group 2 (*n* = 18)	7.11 (2.14)			
Conduct Problems	Group 1 (*n* = 74)	2.45 (1.90)	−1.326	0.188	−0.348
	Group 2 (*n* = 18)	3.11 (1.97)			
Hyperactivity–Inattention	Group 1 (*n* = 74)	4.26 (2.25)	−1.454	0.149	−0.382
	Group 2 (*n* = 18)	5.11 (2.19)			
Peer Problems	Group 1 (*n* = 74)	3.70 (2.33)	−0.501	0.618	−0.132
	Group 2 (*n* = 18)	4.00 (1.94)			
Prosocial Behaviors	Group 1 (*n* = 74)	8.11 (1.72)	−0.871	0.386	−0.229
	Group 2 (*n* = 18)	8.50 (1.69)			
Total Difficulties	Group 1 (*n* = 74)	14.85 (6.98)	−2.513	0.014	−0.661
	Group 2 (*n* = 18)	19.33 (5.87)			
Total Impact	Group 1 (*n* = 74)	2.30 (2.92)	−1.656	0.101	−0.435
	Group 2 (*n* = 18)	3.56 (2.75)			
BDI Total	Group 1 (*n* = 74)	16.65 (12.45)	−3.659	<0.001	−0.962
	Group 2 (*n* = 18)	28.67 (12.61)			
STAI subscales					
State-Anxiety	Group 1 (*n* = 74)	45.53 (12.76)	−3.931	<0.001	−1.033
	Group 2 (*n* = 18)	58.33 (10.68)			
Trait-Anxiety	Group 1 (*n* = 74)	45.22 (12.66)	−3.529	<0.001	−0.927
	Group 2 (*n* = 18)	56.67 (10.90)			

Note: SDQ: Strengths and Difficulties Questionnaire; BDI: Beck Depression Inventory; STAI: State-Trait Anxiety Inventory.

## Data Availability

Raw data will be uploaded on www.zenodo.org immediately after the acceptance of the manuscript and they will be available upon a reasonable request to the authors A.G.U. and A.S.
